# Fast and simple super-resolution with single images

**DOI:** 10.1038/s41598-022-14874-8

**Published:** 2022-07-04

**Authors:** Paul H. C. Eilers, Cyril Ruckebusch

**Affiliations:** 1 Department of Biostatistics, Erasmus MC, Rotterdam, The Netherlands; 2 LASIRE CNRS, University of Lille, Lille, France

**Keywords:** Imaging techniques, Microscopy

## Abstract

We present a fast and simple algorithm for super-resolution with single images. It is based on penalized least squares regression and exploits the tensor structure of two-dimensional convolution. A ridge penalty and a difference penalty are combined; the former removes singularities, while the latter eliminates ringing. We exploit the conjugate gradient algorithm to avoid explicit matrix inversion. Large images are handled with ease: zooming a 100 by 100 pixel image to 800 by 800 pixels takes less than a second on an average PC. Several examples, from applications in wide-field fluorescence microscopy, illustrate performance.

## Introduction

There is a fundamental limit to the spatial resolution of optical microscopy, due to the diffraction of light. A sharp point source will appear smeared out and have the shape of the point spread function (PSF). Interpreting an observed digital scene as a very fine grid of point sources, a digital microscope image will be a discrete convolution of these sources with the PSF, which is often approximated by a Gaussian function.

A major goal of most developments in microscopy is to improve the spatial resolution, from fluorescence imaging^1^ to electron microscopy^2^ with applications in clinical imaging^3^ or plant cell imaging^4^, among others. In particular, the development of fluorescent-labeling techniques has led to spectacular breakthroughs over the past two decades, notably with super-resolution optical microscopy techniques based on stochastic switching of single-molecule fluorescence (PALM^5^, STORM^6^, and SPIDER^7^). These sub-diffraction-limit imaging techniques rely on digital image acquisition and deconvolution algorithms capable of localizing individual fluorescent probes in thousands of image frames and projecting the deconvolved images into a single plane.

However, not all situations are amenable to super-resolution fluorescence imaging, because of computational limitations, or because one does not have access to advanced optical instruments and experienced personnel. Fast and efficient algorithms for resolution enhancement of conventional (fluorescence) images remain highly desirable^8^. Examples include interpolation techniques^9^, learning-based algorithms^10^ and reconstruction approaches based on deconvolution and regularization^11^.

Modern computer languages like Matlab, R and Python provide efficient matrix operations that make it easy to implement convolution with little programming. Deconvolution is the inverse operation, estimating a source image from the observed image. It is a so-called ill-conditioned problem, but it can be tamed by using constraints, implemented as penalties^1,12^.

Here we present an algorithm that works on arbitrary single images, and is very fast: zooming a 100 by 100 pixel image to 800 by 800 pixels takes less than a second on an average PC. The algorithm, which we call TurboZoom, can be coded in 30 lines of Matlab code.

In the next section we describe the theoretical basis of fast deconvolution by ridge regression and how to implement it efficiently with the conjugate gradients algorithm. “Applications” section presents some typical applications. In the final section we discuss options for further research.

## Convolution and deconvolution

In this section, we present the methods and algorithms.

### Convolution and matrix algebra

We assume the following model for an image under study. A latent high-resolution source image is convolved with a high-resolution PSF to form a latent blurred high-resolution image, from which the pixels of the observed low-resolution image are obtained by averaging. Also we assume that the two-dimensional PSF is a radially symmetric Gaussian. This allows us to write it as an outer product of one-dimensional PSFs:

$$\begin{aligned} g(u, v) = c \exp (-(u^2 + v^2)/\sigma ^2) = c \exp (-\rho ^2/\sigma ^2) = c \exp (-u^2/\sigma ^2)\exp (-v^2/\sigma ^2), \end{aligned}$$

where $$\rho ^2 = u^2 + v^2$$.

We first explore one-dimensional convolution and deconvolution. Let *x*, a vector of length *n*, be a latent high-resolution “image”, it has been convolved by a one-dimensional PSF *h* to form a latent image *z*. We observe the vector *y*, of length *m*, which is obtained by averaging groups of *r* “pixels” of *z*. Hence $n = rm$. We can write $z = Hx$, where *H* is the convolution matrix, the columns of which are shifted copies of *h*. We can write the averaging as $y = Cz$, where *C* is an *m* by *n* matrix. In row *i* of *C*, the elements $r(i-1) + 1$ to *ri* are equal to 1/*r*, while all other elements in that row are zero. With $S= CH$, we have that $y=Cz = CHx = Sx$

### Deconvolution with ridge regression

Deconvolution is the inverse of convolution. If $y = Sx$ and *S* and *y* are given, the goal is to determine *x*. Because *x* has more elements than *y*, this is a so-called singular problem: no unique estimate ${\hat{x}}$ can be found. A simple and effective remedy is provided by ridge regression. The objective function is $Q = |y - Sx|^2 + \kappa |x|^2$, where $|.|^2$ indicates the squared norm; e.g. $|x|^2 = \sum _j x_j^2$. Here $\kappa$, a positive number, is the regularization parameter. Increasing $\kappa$ decreases the norm of ${\hat{x}}$, shrinking the solution towards zero.

**Figure 1:**
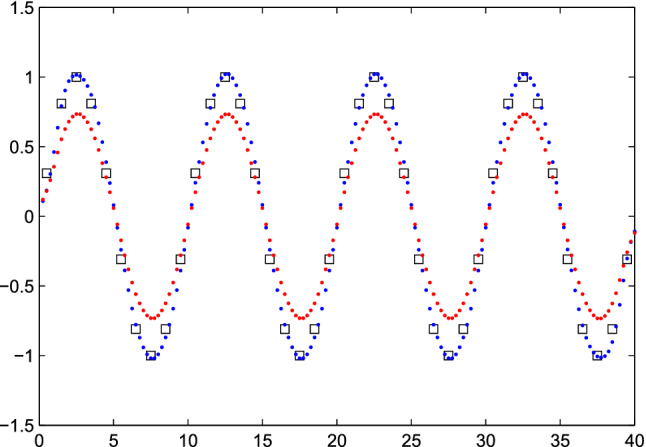
Deconvolution of a sine wave with fourfold super-resolution. The squares show the “observed” series. The blue and red dots represent the estimated ${\hat{x}}$ for $\kappa = 0.01$ and $\kappa = 0.1$.

It is simple to find the ${\hat{x}}$ that minimizes *Q*, because an explicit formula is available: ${\hat{x}} = (S'S + \kappa I) ^{-1}S'y$, with *I* the identity matrix. As Fig. 1 shows, the best results were obtained with $\kappa = 0.01$ and, with $\kappa < 0.01$, essentially the same results are obtained. Only for very low values, less than $10^{-10}$, instabilities occur. For $\kappa = 0.1$ the estimate of *x* is strongly shrunk towards zero. Larger values of $\kappa$ will lead to even more shrinkage. For $\kappa =0$, Matlab reports a solution, but warns that the system is singular, and produces a result that looks more or less random.

### The ringing artifact

**Figure 2:**
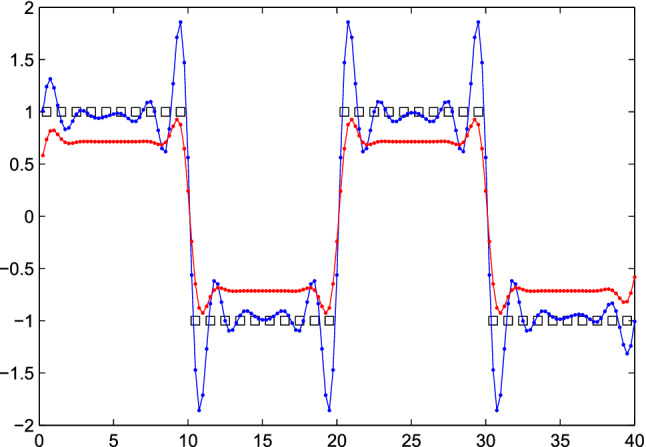
Fourfold super-resolution of a simulated step wave, demonstrating overshoot (ringing). The squares show the “observed” series. The blue and red dots represent the estimated ${\hat{x}}$ for $\kappa = 0.01$ and $\kappa = 0.1$. They have been connected by lines for clarity.

It may seem that small values of $\kappa$ are safe choices, but unfortunately that is not always true. The smoothness of the sine function in Fig. 1 masks a potential artifact. In Fig. 2, *y* shows sharp jumps, leading to overshoot in ${\hat{x}}$. It gets smaller with increased $\kappa$, but it does not disappear. In the engineering literature this is often called “ringing”.

An effective solution is to add an extra penalty, on the “roughness” of *x*, as measured by the differences between neighboring elements. The objective function is changed to

1 $$\begin{aligned} Q = |y - Sx|^2 + \kappa |x|^2 + \lambda |Dx|^2 = \sum _i(y_i - \sum _j s_{ij} x_j)^2 + \kappa \sum _j x_j^2 + \lambda \sum _j (x_j - x_{j-1})^2. \end{aligned}$$

Here $\lambda$ determines the weight of the roughness penalty and *D* is a matrix that forms first differences. The difference penalty is familiar from the Whittaker smoother^13^. As an illustration, *D* is given below if *x* has five elements.

2 $$\begin{aligned} D = \begin{bmatrix} \begin{array}{rrrrr} -1 &{} 1 &{} 0 &{} 0 &{} 0 \\ 0 &{} -1 &{} 1 &{} 0 &{} 0 \\ 0 &{} 0 &{} -1 &{} 1 &{} 0 \\ 0 &{} 0 &{} 0 &{} -1 &{} 1 \end{array} \end{bmatrix}. \end{aligned}$$

**Figure 3:**
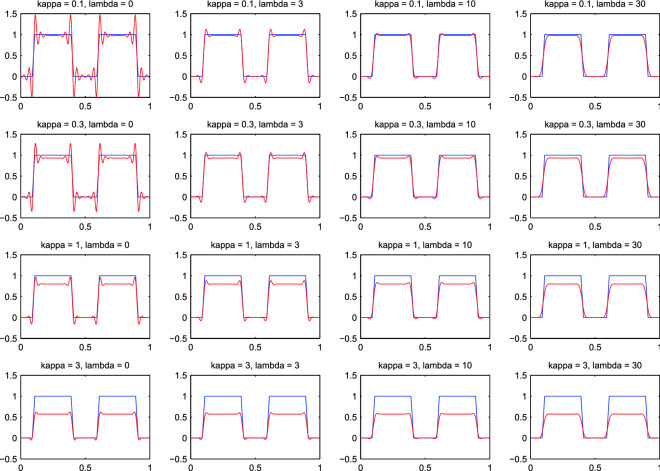
Fourfold super-resolution of a simulated step wave for several combinations of $\kappa$, the ridge parameter and $\lambda$, the smoothness parameter. The blue curve is the true input signal and the red curve its reconstruction.

Figure 3 shows the results of penalized deconvolution for a series of values of $\kappa$ and $\lambda$. With a heavy ridge penalty the overshoot is reduced, but too much shrinking towards zero occurs. With the extra difference penalty, a light ridge penalty is allowed with little ringing.

## Two-dimensional deconvolution

For images we have to deal with two-dimensional convolution, transforming an input image *X* (an *n* by ${\tilde{n}}$ matrix) to the output image *Y* (an *m* by ${\tilde{m}}$ matrix). There are two ways to accomplish this computationally. One is to stack the columns of *X* to make an ($n{\tilde{n}}$) vector $\breve{x}$ and the columns of *Y* to make a vector $\breve{y}$ of length $m{\tilde{m}}$. It is easy to show that $\breve{y} = ({\tilde{S}}\otimes S) \breve{x}$, where ${\tilde{S}}$ is the matrix to do convolution of vectors with ${\tilde{n}}$ elements. Note that ${\tilde{S}}\otimes S$ is a large matrix. Suppose that we want a fourfold increase of the resolution of an image of 100 by 100 pixels. Then ${\tilde{S}}\otimes S$ has $400^2 =160{,}000$ rows and $100^2 = 10{,}000$ columns.

In two dimensions, working with vectorized images and only a ridge penalty, the objective function is

3 $$\begin{aligned} Q = |\breve{y} - ({\tilde{S}} \otimes S) \breve{x}|^2 + \kappa |\breve{x}|^2. \end{aligned}$$

However, it is inefficient to follow this route. In “The conjugate gradients algorithm” section we introduce the conjugate gradients algorithm, that avoids the explicit solution of a system of linear equations. It also allows us to work with images as matrices, avoiding vectorization.

We can avoid vectorizing *Y* to $\breve{y}$ and transforming $\breve{x}$ back to a matrix, because $Y = SX{\tilde{S}}'$ gives the desired result directly. This gives an enormous reduction of storage space and computation, because ${\tilde{S}}\otimes S'$ is not computed at all. The equation $Y = SX{\tilde{S}}'$ can be interpreted as $Y = (S X){\tilde{S}}'$, or as first convolving the columns of *X* with *S*, followed by convolution of the rows of the result with ${\tilde{S}}'$. Similarly $Y = S (X {\tilde{S}}')$ says to first convolve the rows of *X* with ${\tilde{S}}$ and the result with *S*. The final result does not depend on the order of the multiplications

**Figure 4:**
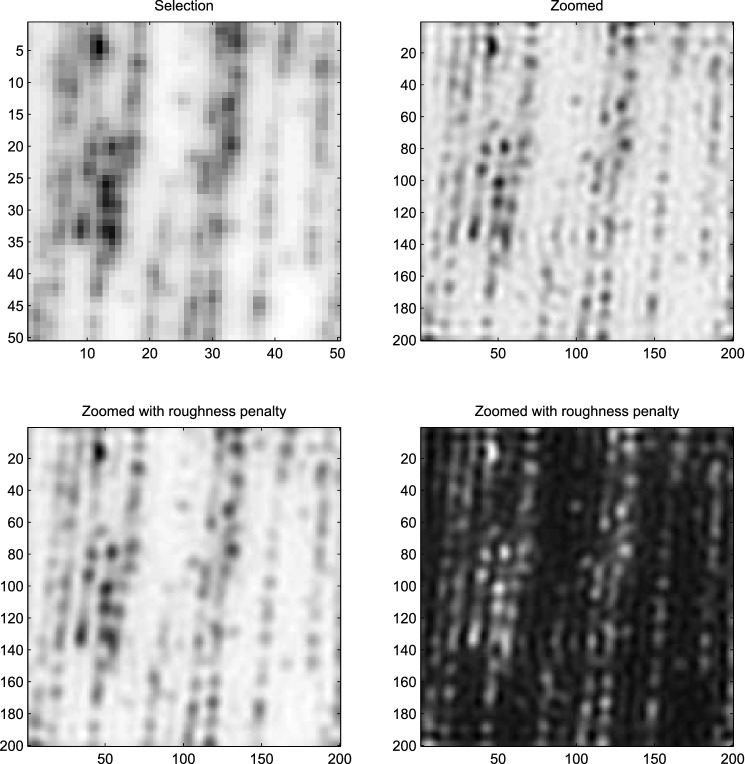
Ringing artifacts in image deconvolution. Upper-left: original image. Upper-right: fourfold super-resolution by deconvolution with $\kappa = 0.001$; ringing is visible. Lower-left: super-resolution with additional roughness penalty with $\lambda = 1$. Images displayed as negatives, except in the lower-right panel.

In two dimensions the ringing artifact appears as spatial ripples. Figure 4 shows an example, using an image of stretched DNA (see “DNA mapping” section) . The image reminds us of heavy raindrops falling into a pond.

We have two sets of difference penalties: one with a penalty on every row of *X* and the other with a penalty of every column. The former can be compactly written as $|DX|^2_F$, and the latter as $|X{\tilde{D}} |^2_F$ where $|.|^2_F$ stands for the square of the Frobenius norm of a matrix, i.e. the sum of the squares of all its elements. Here *D* is the $n - 1$ by *n* differencing matrix and ${\tilde{D}}$ an ${\tilde{n}} - 1$ by ${\tilde{n}}$ differencing matrix.

Note that we present images as negatives. It is more common to use positives, but in our opinion the human eye detects details much better in negatives. Compare the upper-right and lower-left panels in Fig. 4. In the positive image the ringing is essentially invisible.

### The conjugate gradients algorithm

In a modern computer language like Matlab, deconvolution of vectors by ridge regression is not challenging. One line of code does the work: X = (S’ * S + kappa * eye(n)) \ (S’ * y). Unless we have very small images this will not work with vectorization of matrices *Y* and *X*. As we have noted before, too much memory space and computing time are needed when we form vectors $\breve{y}$ and $\breve{x}$ and work on them.

Conjugate gradients (CG) is an algorithm for iteratively solving systems of linear equations^14^. It was developed for the standard case with a matrix and vectors. Its key property is that no matrix inversion is used, only products of matrices and vectors. For our application to images, we can adapt CG in such a way that only products of matrices are needed, and that these matrices are never larger than *Y*, *S*, ${\tilde{S}}$ or *X*. The Supplemental Information shows Matlab code for one-dimensional and two-dimensional CG. Here we discuss some key points.

**Figure 5:**
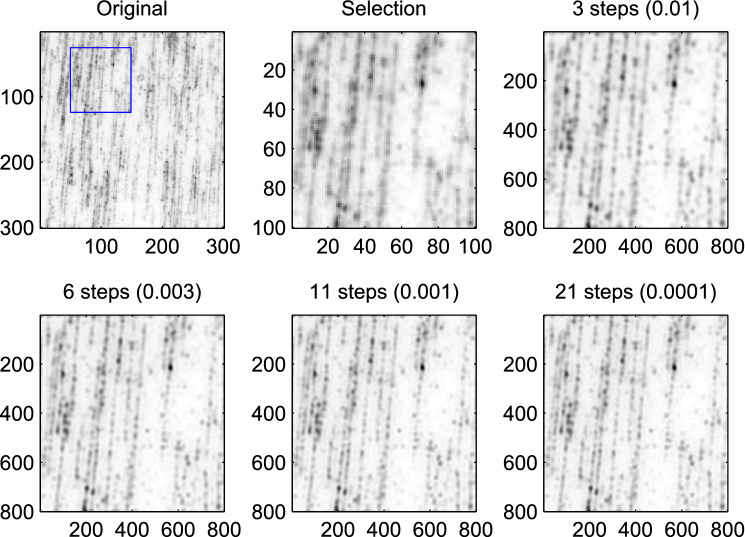
Illustration of the convergence of the conjugate gradients algorithm. The upper-left panel shows a part of an image of stretched DNA (see “DNA mapping” section). The upper-middle panel shows a small selection. The other panels show eight-fold super-resolution images as obtained after increasing numbers of steps. The titles of the panels show the number of iterations and the value of $\delta$, the convergence criterion.

The conjugate gradients algorithm saves memory space, because we can work directly on the matrix *X*. Constructing a classical system of penalized equations would take much time and solving it even more. Another advantage is that we can set the number of iterations at a value that suits our purpose. For initial exploration of images, to locate interesting regions, a handful iterations will be enough. This is illustrated in Fig. 5. We show the core of the code here.

**Listing 1** The core of the conjugate gradients algorithm for solving a linear system.
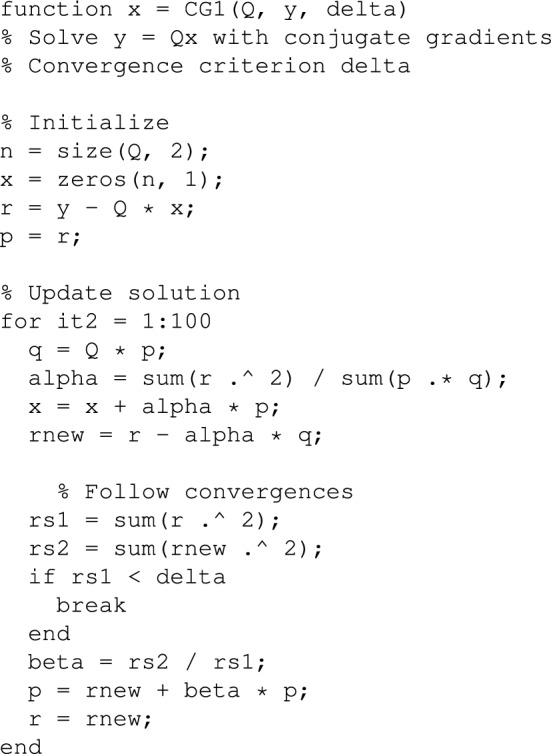


The matrix $G = S' S + \kappa I + \lambda D'D$ and two vectors *p* and *q* are computed beforehand, starting from the observed *y* and setting $x=0$. The iterations update *p*, *q* and *x*. The first line in the loop, computing $q = Gp$, is the most expensive one, but it is relatively cheap, the multiplication of a matrix and a vector.

Of course an iterative algorithm is only useful if it converges quickly enough. In our experience this is the case for conjugate gradients and deconvolution with super-resolution. We illustrate that with Fig. 5, that shows the results obtained for increasing numbers of iterations. In practice ten iterations should be enough. We have not studied convergence in a quantitative way.

## Applications

In this section we present three typical applications. They show images with different characteristics, to illustrate the versatility and effectiveness of our algorithm.

### DNA mapping

Optical mapping is used in many core genomics applications. DNA fragments are labeled with a functionalized dye and stretched on a coated coverslip. The labeled DNA sample is imaged with a wide-field fluorescence microscope (Zeiss Elyra microscope, Plan-APOCHROMAT 63x oil immersion objective, numerical aperture 1.4, EMCCD camera) and a 576 nm excitation laser. The camera pixel size projected in the sample is 80 nm per pixel and the field of view of the image is 24x24 $\mu$m$^2$. More detailed information about sample preparation and image acquisition can be found in^15^.

**Figure 6:**
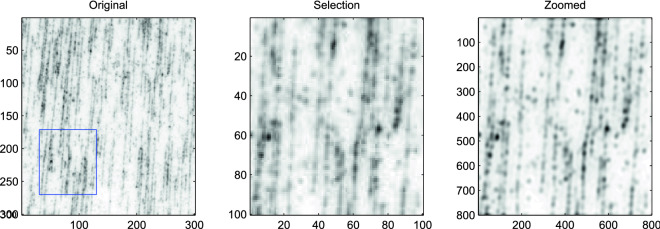
Single image super resolution of the DNA image obtained with a factor 8 up-sampling grid and a PSF FWHM of 400 nm. Left panel: the $300\times 300$ observed image (field of view: $24\times 24$ $\mu$m$^2$) of the linearized labeled DNA fragments. Middle panel: the selected 100 by 100 pixel image. Right panel: super-resolution with a factor 8.

The image in the left panel of Fig. 6 represents alignments corresponding to the positions of the individual emitters associated with the specifically cleaved DNA molecules that were aligned on a surface.

The blue square in the left panel shows the region to which the TurboZoom algorithm was applied; this region is enlarged in the middle panel. The result of super-resolution by a factor 8 is shown in the right panel. The PSF of the microscope was approximated by a two-dimensional Gaussian function with full-width at half-maximum (FWHM) of approximately 350 nm.

Linearized DNA molecules that could hardly be distinguished on the original image can now be clearly resolved. This is of crucial importance as most methods employed for optical mapping of DNA rely on the quality of the signal traces extracted from these images (e.g. for database matching) and require a high accuracy of signal conversion. Another key aspect for DNA high throughput technologies is of course speed. The computation time to obtain the result in Fig. 6 was approximately one second.

### Maize stem cross section

**Figure 7:**
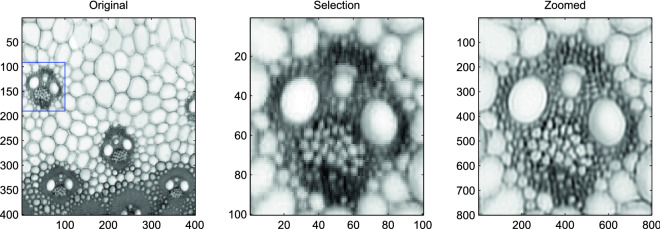
Single image super resolution of the Stem cells image. Left panel: a $400\times 400$ selection of the observed image. Middle panel: the $100\times 100$ pixel selection (indicated by a blue square in the left panel). Right panel: super-resolution with a factor 8.

The image in Fig. 7 was obtained using a macroscopy imaging system to observe the global organization of vascular bundles in cross sections of maize stems. Details about sample preparation and image acquisition can be found in^16^. The left panel of Fig. 7 corresponds to a 1x1 mm$^2$ cross section of the parenchyma of the stem. It allows the cellular morphology of the plant tissues to be quantified.

A factor 8 was set for up-sampling of the original grid. It yielded the reconstructed high resolution image shown in the right panel of Fig. 7. As no information was available about the PSF of the instrument, it was here tuned manually and it was observed that FWHM values in the range 10–15 $\mu$m provide good results; the image shown in Fig. 7 was obtained for a value of 12 $\mu$m. The improvement obtained on the individual vascular bundles and of their distribution within the stem are clear. The visualization and characterization of the individual morphological parameters of the smallest cells of the vascular bundle is usually an issue^16^. With our approach, small bundles of cells, known as xylem, that can be found around the main vessel elements, can be distinguished clearly.

### GATTA cells

**Figure 8:**
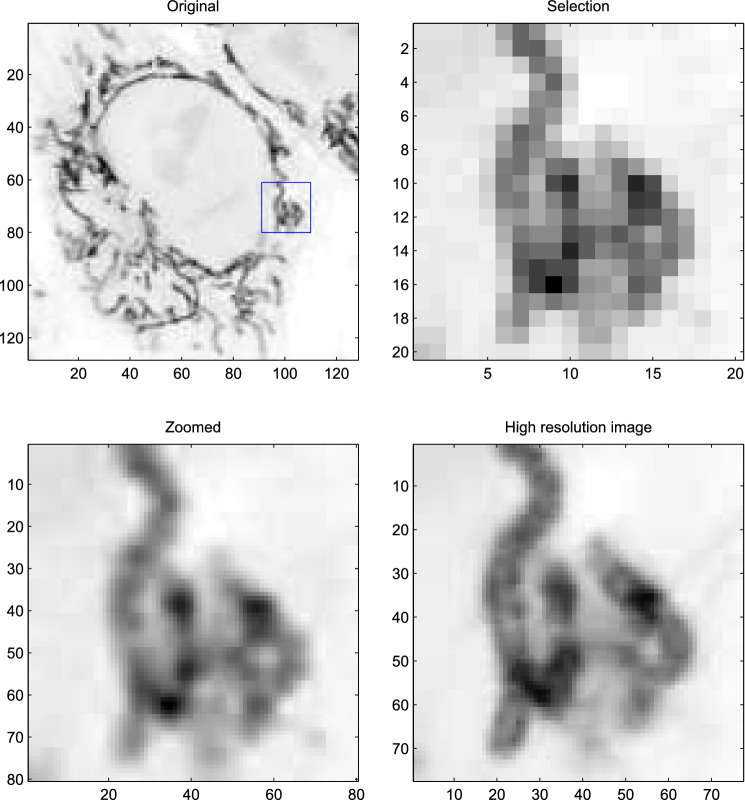
Single image super resolution of GATTA cells. Top-left panel: a $120\times 120$ pixel selection of the observed image. Top-right panel: the $20\times 20$ pixel sub-selection (indicated by the blue square in the top-left panel). Bottom-left panel: super-resolution with a factor 4. Bottom-right panel: the corresponding section of the image observed at four times higher resolution.

GATTA cells (GATTAQUANT GMBH, DNA nanotechnologies) are high-quality standard cell slides which are often used for fluorescence imaging benchmarking in wide field, confocal, and super-resolution microscopy. They contain fixed and stained multicolor huFIB/Cos-7 cells. In the image provided in Fig. 8, only the channel related to the mitochondria (marked with Alexa Fluor 488) was selected, by using adequate fluorescence filters. The image was obtained using a confocal fluorescence microscope (Olympus IX81, Objective, x100, NA 1.4), a 485 nm excitation laser line and a low pass filter with 500 nm cut-off wavelength. The field of view of the image in the left panel of Fig. 8 is 30 by 30 $\mu$m$^2$. More detailed information about the microscopy set up can be found in^17^.

This low-resolution image was acquired on purpose to mimic situations where measurement conditions do not allow the acquisition of a high resolution image. We show the result of super-resolution with a factor 4. The PSF is a two-dimensional Gaussian function with full-width at half-maximum (FWHM) of 200 nm. For comparison, we show four-fold super-resolution with TurboZoom and a true high resolution image. The similarity is strong and no artifacts are visible. Computation takes less than 0.1 s.

The visual impression provided by the reconstructed zoomed image in the bottom-left panel of Fig. 8 is clearly an improvement. The zoom factor was intentionally limited to 4, to compare it to the image in the bottom-right panel, that was obtained with the same microscope, using a four times larger true resolution.

**Figure 9:**
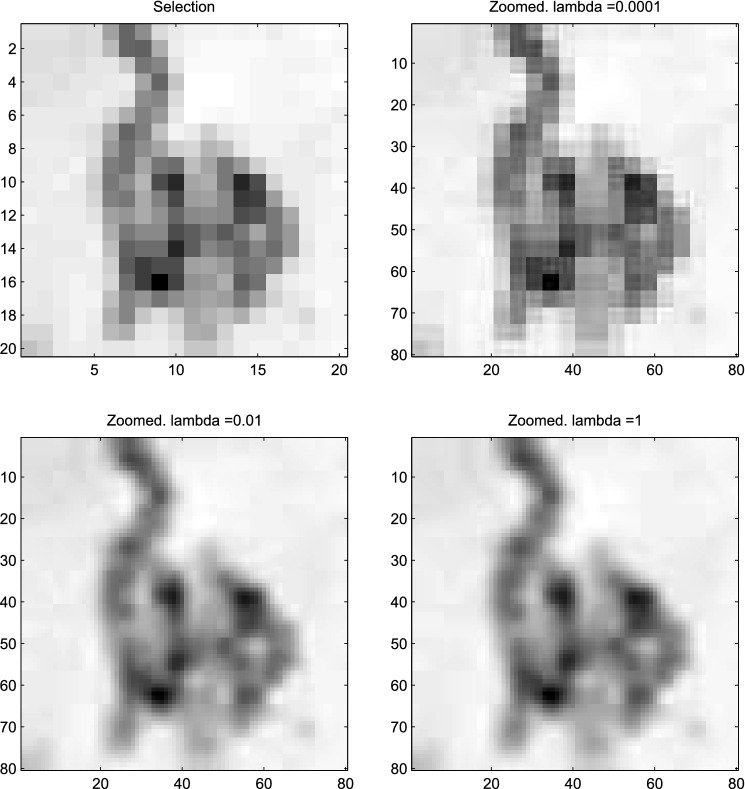
The influence of the roughness penalty. When $\lambda$ is low, pixels of the low-resolution image appear as squares after zooming.

A reviewer pointed out that in the lower-left panel of Fig. 8, the zoomed image shows, in some places, vague blocks of 4x4 pixels that have the same brightness as the pixels in the selected images. It looks as if the original pixels have been blown up to 4x4 patches. We discovered that this effect depends on the value of $\lambda$. Figure 9 shows deconvolutions with widely different values of $\lambda$. We see that the roughness penalty can also serve to smooth discontinuities.

### Contrast enhancement

Some images show spatially varying contrast. We observed that improvements are possible by using asymmetric least squares smoothing. It has been used^7^ for baseline subtraction. The idea is that residuals (the differences between observations and the estimated baseline) below the baseline surface get a small weight (0.01) and those above it a large weight (0.99). Similarly, a “top-line” surface can be estimated by reversing the weights. We subtract the base surface and divide by the difference between top and base surfaces to get a better contrast.

**Figure 10:**
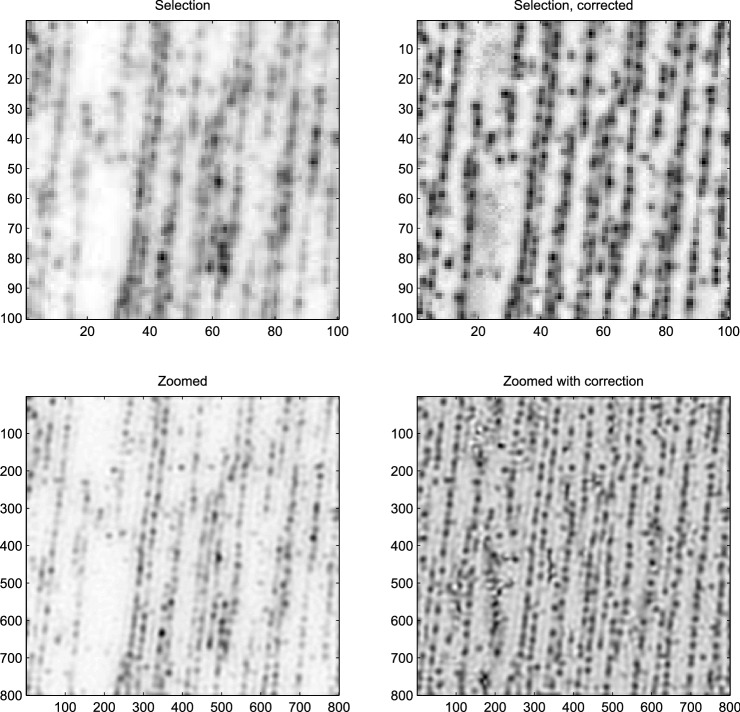
Contrast enhancement with asymmetric smoothing, applied to the DNA image. Upper-left panel: a section of the original image. Upper-right panel: idem, after enhancement. Lower-left panel: zoomed section of the original image. Lower-right panel: idem, after enhancement.

Remarkably, this procedure can lead to a large improvement of our perception of (super-resolution) images. This is illustrated in lower-right panel of Fig. 10, where details appear in the empty regions in the left part of the image.

**Figure 11:**
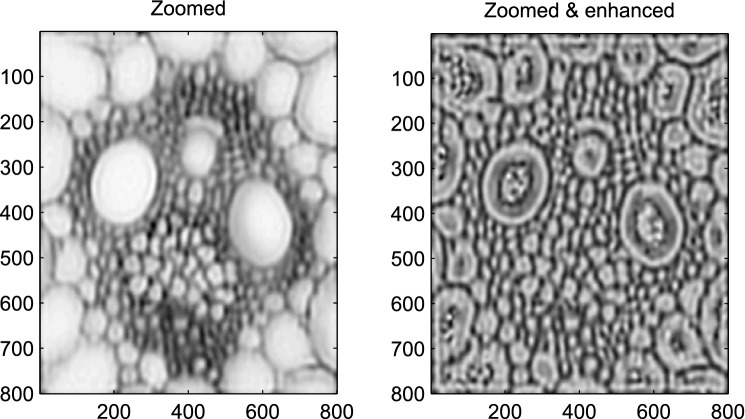
Contrast enhancement with asymmetric smoothing, applies to the stem cross section image. Left panel: zoomed image. Right panel: after enhancement.

A more extreme case is illustrated in Fig. 11, for the stem cell data. The human eye is not good at seeing brightness variation on top of a strong background. By subtracting the background, the absolute variations stay the same, but the relative variations are stronger, leading to better perception. Of course, it depends on the application whether this is useful or not.

We see contrast enhancement as a subjective procedure, improving human perception. Its value depends on several properties of the observed image. It is fast, taking less than a second, so experimenting with it is easy.

## Discussion

We have presented a fast and simple algorithm for resolution improvement. It is based on ridge regression, a familiar tool for solving ill-conditioned estimation problems. There is no need for flashing small molecules, nor for multiple frames; a single image is sufficient. By exploiting the tensor structure of two-dimensional convolution, combined with the conjugate gradients algorithm for (approximately) solving a linear system of equations, fast computation with a small memory footprint is obtained.

Overall, in practical terms, the main features of the proposed approach can be summarized as simplicity, speed and ease of implementation and application. It is easy for the user to tune manually the regularization and PSF parameters, and judge the outcome of the calculations almost instantly.

There are three tuning parameters. One is the width of the point spread function. We expect that users will, from experience, have a good idea of its numerical value. Then there are a shrinking and a smoothing parameter. We do not offer an automatic procedure for choosing their values.

We have not explored quantitative figures of merit. Some of them are only meaningful for localized emitters. We believe that they have received too much attention in recent publications. After all, the part ’scopy’ in the word microscopy tells us that visual exploration of images is paramount.

The objective function in Eq. (3) is a penalized sum of squares. Implicitly it assumes a noise model with constant variance. It is possible to introduce iterative weighting to account for other types of noise, like photon noise.

We have not explored it ourselves, but it is obvious that (massive) parallelization is possible by dividing a given image into a number of rectangles and processing each of them separately. To fix ideas, suppose we have an image with 1000 by 1000 pixels, which we divide into 100 sub-images of 100 by 100 pixels. For an eight-fold improvement in resolution, only one second per sub-image is needed. Assuming a modern workstation with enough processors to deliver ten times the performance of a PC, the work can be done in 10 seconds. For higher speed-ups, graphical processing units can be used. An small experiment with TensorFlow on an NVIDIA GeForce GTX 1650 video card showed tenfold improved performance compared to the CPU of the PC.

The convolution and penalty matrices are sparse, so it seems that there is room for further speed-ups by careful programming. Combining all computing options, it should be possible to achieve close to instant super-resolution.

Although not strictly related to super-resolution, we did pay attention to two “tricks” to improve human perception. In negative images it is easier to detect details than in positive images. In addition we proposed an algorithm for correcting local variations in contrast, based on asymmetric smoothing.

There exists a large literature on single image super-resolution using neural networks. One reviewer suggested to compare our algorithm to one of the best performing implementations^18^. We discovered that this would be a very large project. A large number of images has to be supplied to train the network. In compressed form they already add up to a 7 Gb file. Only moderate up-scaling factors (2 or 4) are considered. Our algorithm needs no training. Only two parameters, for the ridge and the roughness penalty, need tuning. This can be done quickly by visual feedback.

An interesting competitor to our proposal is the work by Zhao et al.^11^. It is fast and Matlab code is available. It uses a special interpretation of the way a low-resolution image was formed: as picking every second, fourth etc. row and columns of pixels of an underlying high-resolution image. Our model assumes averaging groups of 2x2, 4x4 etc. pixels.

Zhao et al.^11^ investigated modified algorithms to allow sparse super-resolution. The key is to replace the sums of squares by the sum of absolute values (L_1_ norm) in the ridge penalty. In addition an L_1_ norm can be used in the difference penalties to obtain sharp edges. It will be interesting to explore if these modifications can be combined efficiently with the conjugate gradients algorithm.

## Supplementary Information

Supplementary Information.

## Data Availability

The data and Matlab programs that support the findings of this study are available in the Git repository https://bitbucket.org/PaulEilers/turbozoomrepo.
