# Supplementary material for: Fast and simple super-resolution with single images
Source: Sci Rep. 2022 Jul 4;12:11241. doi: 10.1038/s41598-022-14874-8 (PMC9253020; doi:10.1038/s41598-022-14874-8)
Supplement: Supplementary file 1 — Supplementary Information. [file 41598_2022_14874_MOESM1_ESM.pdf]

Supplementary Information for Fast and Simple Super-resolution with Single Images

Paul H.C. Eilers Cyril Ruckebusch

May 23, 2022

Here we present details of the conjugate gradients (CG) algorithm and its Matlab implementation, based on the Wikipedia lemma (`https://en.wikipedia.org/wiki/Conjugate_gradient_method`). We start with the one-dimensional case. The PSF is represented by the m-by-n matrix S and the observed "image" is the m-vector y. We have to solve the system of penalized least squares equations $(S'S + \kappa I + \lambda D'D)x = S'y$ for the n-vector x. For efficiency, it is re-written as $Gx = u$.

Listing 1: Code for standard conjugate gradients algorithm.

```
% Set up ridge regression
n = size(S, 2);
kappa = 0.001;
D = diff(eye, n);
lambda = 1;
G = S' * S + kappa * eye(n) + lambda * D' * D;
u = S' * y;

% Initialize for conjugate gradients
x = zeros(n, 1);
r = u - G * x;
p = r;

% Iterate CG
for it = 1:50

% Update p and r
```

```
q = G * p;
alpha = (r' * r) / (p' * q);
x = x + alpha * p;
rnew = r - alpha * q;
beta = (rnew' * rnew) / (r' * r);
r = rnew;
p = r + beta * p;

% Monitor convergence
err = sqrt(mean((u - G * x) .^ 2 ));
disp([it log10(err)])
if err < 1e-3
break
end
end
```

The core of the algorithm takes only seven lines. One operation is a matrix-vector product (`q = G * p`), all others work on only vectors.

Listing 2: Code for two-dimensional conjugate gradients algorithm.

```
% Prepare components of linear system
U = S1' * Y * S2;
G1 = S1' * S1;
G2 = S2' * S2;
kappa = 1;
lambda = 1;
D1 = diff(eye(n1));
D2 = diff(eye(n2));
V1 = lambda * D1' * D1;
V2 = lambda * D2' * D2;

% Initialize for conjugate gradients
R = U;
P = R;
n1 = size(G1, 2);
n2 = size(G2, 2);
X = zeros(n1, n2);

for it = 1:100
```

```
% Update P and R
Q = G1 * P * G2 + kappa * P + V1 * P + P * V2';
alpha = sum(R(:) .^ 2) / sum(P(:) .* Q(:));
X = X + alpha * P;
Rnew = R - alpha * Q;
rs1 = sum(R(:) .^ 2);
rs2 = sum(Rnew(:) .^ 2);
beta = rs2 / rs1;
P = Rnew + beta * P;
R = Rnew;

% Monitor convergence
rms = sqrt(rs1 / (n1 * n2));
disp([it log10(rms)])
if rms < 1e-3
break
end
end
```
